# *QTL*-Guided Prioritization of *SiMGT2* as a Candidate Gene Associated with Grain Fat Content in Foxtail Millet

**DOI:** 10.3390/biology15181631

**Published:** 2026-09-16

**Authors:** Wei Zhou, Hongyuan Yang, Hui Zhi, Cheng Zhang, Honggang Zhang, Minghui Guo

**Affiliations:** 1 Shanxi Institute for Functional Food, HouJi Laboratory of Shanxi Province, Shanxi Agricultural University, Taiyuan 030031, China; nx06114@163.com (W.Z.);; 2 Shanxi Center for Inspection and Testing, Shanxi Institute of Standardization and Metrology Technology, Taiyuan 030006, China; yanghongyuan9186@163.com; 3 State Key Laboratory of Crop Gene Resources and Breeding, Key Laboratory Grain Crop Genetic Resources Evaluation and Utilization, Institute of Crop Sciences, Chinese Academy of Agricultural Sciences, Ministry of Agriculture and Rural Affairs, Beijing 100081, China

**Keywords:** foxtail millet, magnesium transporter, *QTL* mapping, genome-wide analysis, haplotype analysis

## Abstract

Grain fat content contributes to nutritional quality in foxtail millet, but its genetic basis remains incompletely understood. We evaluated a 256-line recombinant inbred population derived from Jingu21 and Chuang29 in two environments. Among the individual environments, *qFC5* was detected only in Sanya (2022_SY; LOD 5.86; phenotypic variance explained: 9.24%), exceeding the LOD threshold of 3.58. Across-environment mapping using best linear unbiased estimates identified an overlapping chromosome 5 region (LOD: 5.99; phenotypic variance explained: 7.67%), exceeding the threshold of 4.14 from 1000 permutations. Among 248 annotated genes in the 1.80 Mb Sanya interval, *Arabidopsis* and rice homology, functional annotation and expression information supported prioritization of *Seita.5G394800.1* (*SiMGT2*), a predicted MGT/MRS2 magnesium transporter. Six SiMGT family members were identified. In the parental panicle dataset, *SiMGT2* had a higher mean expression in Jingu21 at three sampling stages; only the middle-stage difference remained significant after correction across 18 comparisons. In 534 phenotyped accessions, H1 had a higher grain fat content than H2, but not H3; these comparisons were not corrected for population structure or kinship. The results prioritize *SiMGT2* as a candidate associated with grain fat content, with environment-dependent *QTL* support. Causality and the proposed connection with magnesium homeostasis require functional validation.

## Simple Summary

Grain fat content contributes to the nutritional and processing qualities of foxtail millet. We examined a population derived from Jingu21 and Chuang29 in two environments and identified a chromosome 5 region associated with this trait in Sanya. An analysis combining the two environments supported an overlapping region, but the locus was not consistently detected across environments. Among 248 genes in the Sanya interval, homology, functional annotation and expression information led us to prioritize SiMGT2, a predicted magnesium transporter. Parental expression differences and an exploratory comparison of natural haplotypes provided additional information for candidate selection. These observations do not establish that *SiMGT2* controls grain fat content. Functional experiments and measurements of grain magnesium and lipids are needed before its biological role or breeding value can be established.

## 1. Introduction

Foxtail millet is an ancient C_4_ cereal crop with strong adaptation to drought-prone and marginal environments [1,2,3]. Owing to its agronomic resilience, stable productivity under resource-limited conditions and diverse grain-use potential, foxtail millet has attracted increasing attention as a climate-resilient crop for value-added grain products [4,5]. Grain chemical composition is a key determinant of the utilization potential of foxtail millet, and components such as starch, protein, fat, dietary fiber, minerals and bioactive compounds contribute to its compositional quality, processing characteristics and value-added properties [6,7]. Among these components, grain fat content is an important lipid-related quality trait because lipids contribute to the chemical composition and energy value of millet grains and may influence processing- and storage-related quality through lipid-derived compounds [5,8]. However, compared with major agronomic traits, the genetic basis of grain fat content in foxtail millet remains insufficiently understood.

Genetic mapping has become an important strategy for dissecting complex traits and identifying breeding-relevant loci in foxtail millet. Population resequencing and genome-wide association studies have revealed extensive genomic variation and loci associated with agronomic traits in diverse foxtail millet panels [9,10,11], while high-density linkage maps and *QTL* mapping in biparental populations have enabled the detection of loci controlling plant height, panicle architecture, yield components and metabolite-related traits [12,13]. These studies have greatly advanced genetic dissection and candidate-gene discovery in foxtail millet. However, most reported loci have focused on agronomic and yield-related traits, whereas the genetic basis of grain chemical composition, particularly lipid-related grain composition, remains less well characterized. Recent studies have revealed substantial variation in foxtail millet grain components, including protein, starch, fat, minerals and bioactive compounds, but the genomic regions and candidate genes underlying grain fat content remain limited [14,15]. Therefore, identifying *QTLs* and candidate genes associated with grain fat content is important for understanding the genetic basis of lipid-related grain composition and for developing value-added foxtail millet germplasm.

Magnesium (Mg^2+^) is an essential mineral element involved in photosynthesis, Mg-ATP-dependent metabolism, enzyme activation, membrane function and ion homeostasis in plants [16,17]. These processes are closely linked to carbon assimilation, energy supply and seed development, suggesting that Mg^2+^ homeostasis may influence the metabolic environment required for the accumulation of grain storage compounds. In higher plants, Mg^2+^ transport is mediated by several transport systems, among which the CorA-type MGT/MRS2 family represents one of the most conserved magnesium transporter groups [18,19]. Typical MGT/MRS2 proteins contain a conserved C-terminal region with two transmembrane helices and the characteristic GMN motif, which is important for Mg^2+^ selectivity and transport activity [18,20,21]. Genome-wide characterization of MGT/MRS2 family members has been reported in Arabidopsis, rice, maize, rapeseed and tomato, revealing conserved structural features, evolutionary diversification and tissue- or stress-responsive expression patterns [18,19,22,23,24]. However, the MGT/MRS2 family in foxtail millet remains insufficiently characterized, and whether any MGT member is associated with loci controlling lipid-related grain composition, particularly grain fat content, has not been explored.

Here, MGT/MRS2 denotes the plant magnesium-transporter family related to bacterial CorA and yeast Mrs2 proteins; CorA-like describes that sequence and structural relationship, and *SiMGT1*–*SiMGT6* are the foxtail millet gene names. We hypothesized that a transporter candidate within a grain-fat *QTL* could be associated with grain composition through Mg-dependent physiological processes. Whether such a connection exists in foxtail millet, and whether it reflects causal transporter activity, remains an unresolved knowledge gap.

In this study, a recombinant inbred line population derived from Jingu21 and Chuang29 was used to map *QTLs* associated with grain fat content in foxtail millet. A *QTL* on chromosome 5, designated as *qFC5*, was identified, and *Seita.5G394800.1*, encoding a CorA-like magnesium transporter and designated as *SiMGT2*, was located within the *QTL* interval. To evaluate *SiMGT2* within the broader context of the MGT/MRS2 family, we performed a genome-wide characterization of MGT/MRS2 magnesium transporter genes in foxtail millet, including analyses of chromosomal distribution, phylogenetic relationships, conserved motifs, gene structures, promoter cis-acting elements, syntenic relationships and evolutionary rates. Panicle RNA-seq data from Jingu21 and Chuang29 were further analyzed to compare the expression patterns of *SiMGT* genes during panicle development. In addition, haplotype association analysis was conducted in a foxtail millet diversity panel to evaluate the relationship between allelic variation in *SiMGT2* and grain fat content. By integrating *QTL* mapping, gene family characterization, comparative expression analysis and haplotype association, this study prioritizes *SiMGT2* as a *QTL*-co-localized candidate gene associated with grain fat content and provides a basis for further investigation of the relationship between magnesium transport and lipid-related grain composition in foxtail millet.

## 2. Materials and Methods

### 2.1. Plant Materials and Phenotypic Evaluation

A recombinant inbred line (RIL) population consisting of 256 lines derived from a cross between Jingu21 and Chuang29 was used in this study. The RIL population and the two parental lines were grown in 2022 at two field locations: Sanya, Hainan Province, China, during the winter growing season from November to March, and Yuci, Shanxi Province, China, during the summer growing season from May to October. These two field trials were treated as independent environments for phenotypic evaluation and *QTL* analysis of grain fat content.

At each location, genotypes were planted in 2 m row plots with 30 cm within-row plant spacing and received uniform local agronomic management. At physiological maturity, panicles of plants with similar maturity and growth in the central portion of each row were collected and air-dried under the same conditions. This common spatial sampling criterion reduced border effects, although selection for uniform growth could introduce sampling bias. Grains were mechanically dehulled before measurement. At least 200 g of dehulled grain was used in the measurement workflow. Each sample was scanned three times, and the technical scans were averaged to obtain one sample-level estimate. Repeated scans were not counted as independent biological replicates.

Grain fat content was estimated using a DA 7200 near-infrared reflectance analyzer (Perten Instruments AB, Hägersten, Sweden) and the cereal/grain fat model available in the instrument system. The supplied model validation statistics were R^2^val = 0.88, SEP = 0.18 and RPD = 2.0. These described the calibration model and were not an independent validation of the present foxtail millet samples. The phenotype was, therefore, described as NIR-estimated grain fat content, expressed as dry-weight percentage according to the measurement records.

Descriptive statistics and coefficients of variation (CV = SD/mean × 100%) were calculated separately by environment. An approximate entry-mean broad-sense heritability was calculated as H^2^ = σ^2^G/[σ^2^G + σ^2^R/e], where σ^2^G = (MSG − MSR)/e, σ^2^R = MSR and e = 2. Unequal sample replication and pooled residual variation limit interpretation of this estimate.

### 2.2. QTL Mapping for Grain Fat Content and Candidate Gene Identification

The high-density bin map of the Jingu21 × Chuang29 RIL population, previously reported by Zhou et al. [25], was used for *QTL* mapping in this study. Briefly, this map contained 3451 bin markers generated from 372,243 high-quality SNPs using a sliding-window approach. Environment-specific RIL means were analyzed separately for 2022_SY and 2022_YC, each using 1000 permutations at α = 0.05 to determine experiment-wise LOD thresholds. Across-environment best linear unbiased estimates (BLUEs) were obtained from y_ij_ = μ + G_i_ + E_j_ + ε_ij_, with genotype fitted as fixed and environment as random, using restricted maximum likelihood. BLUEs were then used for *QTL* mapping. The genome-wide significance threshold for the BLUE analysis was 4.14, based on 1000 permutations at α = 0.05. While the BLUE-based mapping provides a robust across-environment estimate of *QTL* effects, it does not formally test the *QTL* × environment interaction.

All 248 annotated genes in the 2022_SY *qFC5* interval were examined using homologous comparisons with *Arabidopsis* and rice, functional annotations and available developmental expression profiles. Annotations were considered for potential relationships with lipid metabolism, seed development, Mg homeostasis or related processes; expression supplied tissue context rather than a causal criterion. *SiMGT2* was selected qualitatively as a plausible focal candidate; no numerical ranking or criterion based on proximity to the *QTL* peak was applied.

### 2.3. Identification of MGT Family Members in Foxtail Millet

MGT family members were identified from the *Setaria italica* v2.2 genome and annotation release downloaded from DOE-JGI Phytozome (genome ID 312; https://phytozome-next.jgi.doe.gov/info/Sitalica_v2_2; accessed on 10 May 2026). This release and its Seita gene identifiers were used for genomic coordinates, candidate annotation, protein and CDS extraction, promoter analysis and RNA-seq mapping. Profiles PF05653 and PF01544 were obtained from Pfam [26] and searched against the proteome using HMMER v3.3.2 [27], with an E-value cutoff of 1 × 10^−8^.

In parallel, a homology-based search was conducted using *Arabidopsis* MGT protein sequences as queries. Arabidopsis MGT sequences were selected based on previously reported MGT family members [18] and retrieved from The Arabidopsis Information Resource (TAIR) [28]. BLASTP was performed against the *S. italica* protein dataset downloaded from Phytozome using BLAST+ v2.15.0+ [29,30], with an E-value cutoff of 1 × 10^−10^. The resulting hits were further filtered to retain alignments with more than 30% sequence identity and query coverage greater than 0.5, where query coverage was calculated as alignment length divided by query length.

Candidate foxtail millet MGT proteins were identified using both HMMER and BLASTP searches. The candidate sequences obtained from the two approaches were combined, and redundant sequences were removed to generate a preliminary set of putative SiMGT family members. Protein and CDS sequences of the corresponding candidate genes were extracted from the genome annotation files using seqkit based on gene IDs. To reduce false positives, all candidate proteins were further examined by hmmscan against the Pfam-A database, and sequences lacking MGT/MRS2- or CorA-like magnesium transporter-related domain signatures were excluded. Transmembrane helices were predicted using DeepTMHMM (https://dtu.biolib.com/DeepTMHMM; accessed on 16 May 2026) [31], and the conserved GMN motif was inspected by multiple sequence alignment. Candidates containing the conserved MGT/MRS2-related domain, the characteristic GMN motif and the typical transmembrane topology of plant MGT/MRS2 transporters were retained as bona fide SiMGT family members.

### 2.4. Basic Information Analysis and Prediction of Secondary Structure and Subcellular Localization

Chromosomal locations of the foxtail millet MGT genes were obtained from genome annotation files and visualized using ggplot2 (v4.0.0) in R [32]. The molecular weight (MW) and theoretical isoelectric point (pI) of each SiMGT protein were calculated using the R package Peptides (v2.4.6) [33].

The secondary structures of SiMGT proteins were predicted using the SOPMA server with default parameters, including a window width of 17, a similarity threshold of 8 and four conformational states [34]. The proportions of α-helix, extended strand, β-turn and random coil were calculated according to the predicted secondary-structure states. Subcellular localization of SiMGT proteins was predicted using WoLF PSORT (https://wolfpsort.hgc.jp/; accessed on 16 May 2026) with default parameters [35].

### 2.5. Multiple Sequence Alignment and Phylogenetic Analysis

Protein sequences of SiMGT family members were aligned using the msa package (v1.24.0) with the Clustal Omega algorithm [36,37]. Multiple sequence alignments were generated under default settings and exported in Clustal format.

A maximum-likelihood tree was inferred from the aligned MGT protein sequences using IQ-TREE v2.2.3 [38]. ModelFinder selected JTT+G4 according to the Bayesian information criterion [39]. Branch support was assessed using 1000 SH-like approximate likelihood ratio test (SH-aLRT) replicates and 1000 ultrafast bootstrap replicates [40]. The supplied maximum-likelihood topology was displayed as a rectangular cladogram, with internal-node labels reporting SH-aLRT/ultrafast-bootstrap support (%). Branch lengths are not represented by the displayed horizontal distances. The Newick tree is provided as supplementary computational output.

### 2.6. Gene Structure, Conserved Motif and Cis-Acting Element Analyses

Conserved motifs in the SiMGT protein sequences were identified using MEME (v5.5.5). MEME was run with the maximum number of motifs set to 10, and motif widths were constrained to 6–200 amino acids [41]. Gene structure, including exon–intron organization, and conserved motif distribution were visualized using GSDS 2.0 [42].

To identify putative cis-regulatory elements, the 2000 bp upstream sequences of each *SiMGT* gene were extracted from the reference genome as promoter regions. These promoter sequences were submitted to the PlantCARE database for cis-acting element prediction and annotation [43]. The distribution and frequency of annotated cis-elements were summarized and visualized in R using ggplot2.

### 2.7. Collinearity and Evolutionary Rate Analyses

Collinearity analysis was performed to characterize duplication patterns of the *SiMGT* gene family in the foxtail millet genome. Syntenic blocks and collinear gene pairs were identified using MCScanX v1.0.0 [44] with default settings, and the resulting intragenomic collinearity relationships were summarized and visualized based on MCScanX outputs.

To assess selective constraints acting on duplicated *SiMGT* gene pairs and foxtail millet–rice orthologous *MGT* gene pairs, synonymous (Ks) and nonsynonymous (Ka) substitution rates were estimated using KaKs_Calculator v3.0 [45], applying the standard genetic code and the Yang–Nielsen method [46]. The divergence time (T) was inferred from Ks using the following formula:

T (MYA) = [Ks/(2 × 9.1 × 10^−9^)] × 10^−6^

The assumed rate of 9.1 × 10^−9^ substitutions per synonymous site per year follows a previous gene-family analysis in *Populus* [47]; it is not a rate calibrated for *Setaria* or rice. The factor of two represents divergence along two lineages, and 10^−6^ converts years to millions of years. These rate-dependent values are illustrative and are not used to assign precise duplication or species divergence ages. Synonymous-site saturation was not tested.

### 2.8. RNA-Seq Analysis of SiMGT Genes During Panicle Development

Parental plants of Jingu21 and Chuang29 were grown at Yuci, Shanxi Province, in 2025 for an independent panicle RNA-seq experiment. Heading was recorded separately for each parent when approximately 50% of plants showed emergence of about half of the main-stem panicle from the flag-leaf sheath. Developing panicles were collected approximately 7, 14 and 21 days after the respective heading date, designated P-P, P-M and P-L. These were operational sampling times rather than a formal morphological staging system. Three independent biological replicates per parent and stage yielded 18 samples, which were frozen in liquid nitrogen and stored at −80 °C.

Total RNA was extracted and quality-assessed before library construction. Polyadenylated mRNA was enriched using oligo(dT) magnetic beads (Vazyme, Cat. No. N401-01, Nanjing, China) and fragmented. First-strand cDNA was synthesized with random hexamers, followed by second-strand synthesis, end repair, adapter ligation, PCR amplification and size selection. Libraries with an approximately 380 bp insert were checked using an Agilent 2100 Bioanalyzer (Agilent Technologies, Santa Clara, CA, USA) and sequenced on an Illumina NovaSeq platform (Illumina, Inc., San Diego, CA, USA) with paired-end 150 bp reads. Adapter-containing, ambiguous and low-quality reads were filtered from the raw data. Clean reads were aligned to the *Setaria italica* v2.2 reference genome using HISAT2 v2.2.1 with strand-specific alignment settings for reverse-stranded libraries. SAM files were converted to BAM format, sorted and indexed using SAMtools v1.19.2. Gene-level expression was quantified as fragments per kilobase of transcript per million mapped reads (FPKM). For sample-level quality assessment, principal component analysis (PCA) of centered log_2_(FPKM + 1) values and Pearson sample correlations were calculated across variable genes.

The RNA-seq analysis evaluated the six preselected *SiMGT* genes rather than performing transcriptome-wide differential expression discovery. Two-sided Welch’s *t*-tests compared the parents within each stage using log_2_(FPKM + 1), with three biological replicates per group. Benjamini–Hochberg correction was applied jointly across all 18 gene–stage tests.

In addition, publicly available foxtail millet expression data across multiple tissues and developmental stages were obtained from Setaria-db [48]. The FPKM values of the six *SiMGT* genes were extracted and transformed as log_2_(FPKM + 1). The transformed values were subsequently standardized using row-wise z-score normalization and visualized as a heatmap.

### 2.9. Haplotype Variation and Phenotype Association Analysis of SiMGT2

*SiMGT2* haplotypes were examined in the re-sequenced foxtail millet diversity panel described by Jia et al. [9], using the supplied gene-region variant matrix. The three retained haplotypes were represented by 878 accessions; phenotype comparisons used the 534 accessions with available 2013_TY grain fat content. Two-sided Wilcoxon rank-sum tests were implemented as Mann–Whitney U tests with an asymptotic, tie-corrected variance and continuity correction. Benjamini–Hochberg correction was applied across the three comparisons. Effect sizes were reported as differences in mean fat content, in percentage points, with Welch–Satterthwaite 95% confidence intervals. These group comparisons were not adjusted for population structure or kinship.

## 3. Results

### 3.1. Chromosomal Distribution of SiMGT Genes and Co-Localization of SiMGT2 with qFC5

Jingu21 had a higher NIR-estimated grain fat content than Chuang29 in both environments: 6.24% versus 5.29% in 2022_SY and 5.58% versus 4.59% in 2022_YC. The RIL means were 5.30 ± 0.31% in 2022_SY (*n* = 253; range: 4.18–6.12%; CV: 5.93%) and 4.87 ± 0.32% in 2022_YC (*n* = 232; range: 3.71–5.66%; CV: 6.48%). Distributions were continuous; 125 RILs were below the lower parent in 2022_SY, while 37 were below the lower parent and two exceeded the higher parent in 2022_YC (Table S1; Figure S1). Among 229 RILs with paired observations, the inter-environment correlation was modest (*r* = 0.290; *p* = 8.19 × 10^−6^). Line-mean ANOVA detected genotype (*F_228,228_* = 1.82; *p* = 3.91 × 10^−6^) and environment effects (*F_1,228_* = 274.26; *p* = 5.64 × 10^−41^). The approximate entry-mean heritability was 0.45 under the pooled-residual model; genotype × environment variation could not be separated from line-mean error.

**Figure 1:**
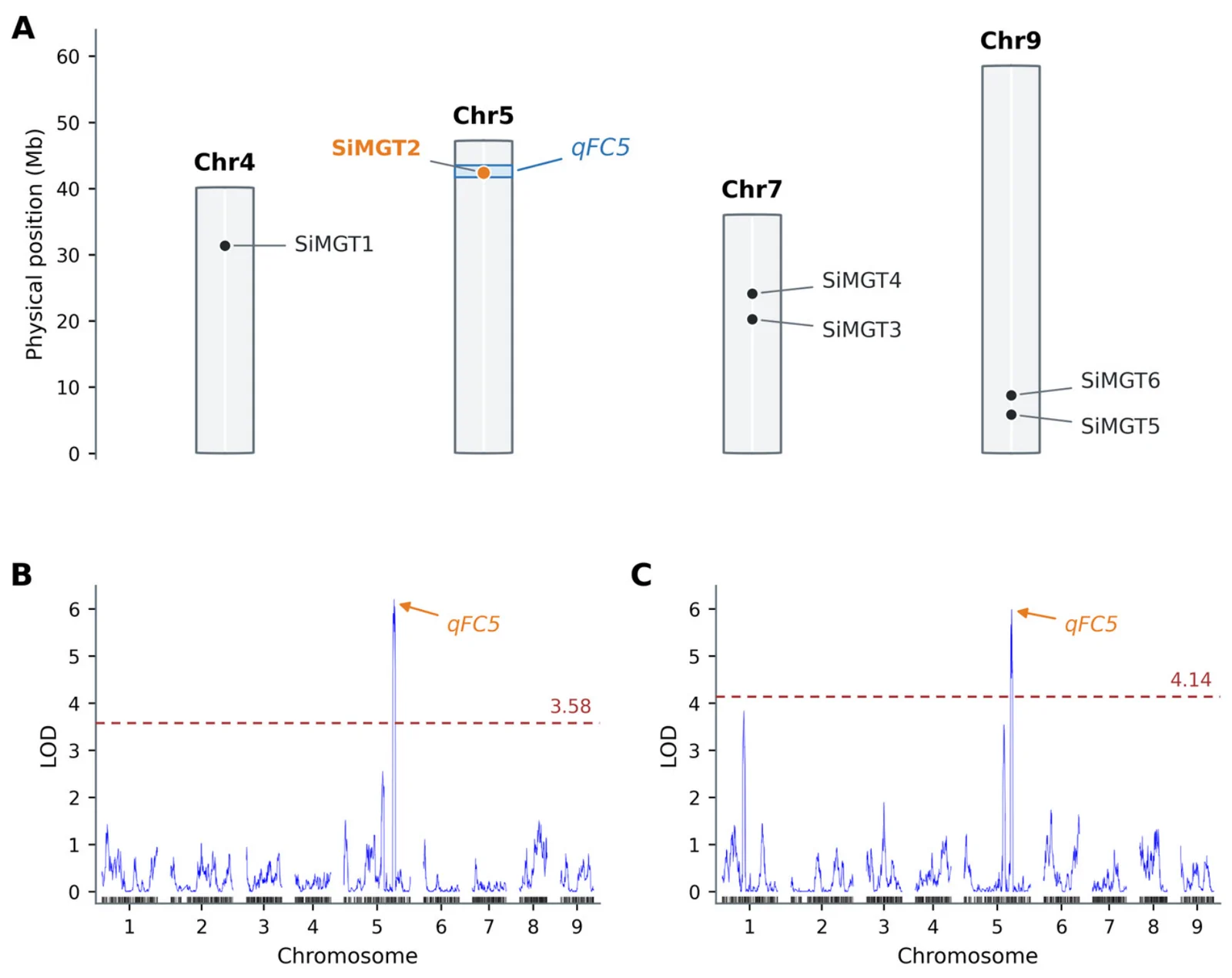
Genome-wide *QTL* profiles, chromosomal distribution of *SiMGT* genes and physical relationship between *qFC5* and *SiMGT2*. (**A**). Physical distribution of the six *SiMGT* genes on foxtail millet chromosomes 4, 5, 7 and 9. (**B**). Genome-wide LOD profile for grain fat content in 2022_SY environment. (**C**). Genome-wide LOD score profile based on best linear unbiased estimates (BLUEs) across environments. The dashed red lines indicate the genome-wide significance thresholds of 3.58 and 4.14, determined by 1000 permutation tests.

In 2022_SY, *qFC5* exceeded the LOD threshold of 3.58, with a peak LOD of 5.86 at marker 42402870_42583113_12 (90.77 cM), a PVE of 9.24% and an additive effect of 0.61. Its 1.5-LOD support interval was 89.48–93.59 cM, corresponding to Chr5:41,697,302–43,493,061 bp. In 2022_YC, the corresponding region on chromosome 5 did not exceed the significance threshold (Figure S2). BLUE-based mapping detected an overlapping region (LOD: 5.99; threshold: 4.14; PVE: 7.67%; additive effect: 0.58), with peak marker 39334859_39420541_6 at 87.84 cM and an interval of 86.01–91.42 cM (Chr5:38,020,311–43,717,324 bp). The increasing allele was contributed by Jingu21. The BLUE and Sanya peaks were not identical; their overlapping intervals provide across-environment support for a chromosome 5 signal but do not establish a stable *QTL* or a common causal variant (Figure 1; Table S2; Figure S2).

The 1.80 Mb *qFC5* interval identified in 2022_SY contained 248 annotated genes, all listed with *Arabidopsis* and rice homolog annotations and public expression profiles in Table S3. *Seita.5G394800.1* (*SiMGT2*), at Chr5:42,398,706–42,401,512 bp, was annotated as a CorA-like magnesium transporter with homologs *AT3G19640* and *LOC_Os01g64890*. Its annotation and expression in panicle-related and grain development categories, together with the parental expression comparison, motivated selection as a biologically plausible focal candidate. This qualitative prioritization does not establish *SiMGT2* as the uniquely best candidate and does not exclude other genes in the interval.

### 3.2. Genome-Wide Identification and Basic Features of SiMGT Genes

To systematically characterize the MGT magnesium transporter family in foxtail millet, genome-wide identification was performed using HMMER- and homology-based searches. A total of six non-redundant *MGT* genes were identified in the foxtail millet genome and designated as *SiMGT1* to *SiMGT6* according to their chromosomal positions (Figure 1; Table 1). These genes were unevenly distributed on four chromosomes, including Chr4, Chr5, Chr7 and Chr9. Chr7 and Chr9 each contained two *SiMGT* genes, whereas Chr4 and Chr5 each harbored one member, indicating a non-uniform chromosomal distribution of the *SiMGT* family (Figure 1). Notably, *SiMGT2*, corresponding to *Seita.5G394800.1*, was the only *SiMGT* member located on Chr5 and was positioned within the *qFC5* interval identified for grain fat content.

The encoded SiMGT proteins ranged from 387 to 457 amino acids in length. Their predicted molecular weights varied from 42.70 to 50.07 kDa, and their theoretical isoelectric points ranged from 4.47 to 5.16, suggesting that all SiMGT proteins are acidic proteins. Transmembrane domain prediction showed that all six SiMGT proteins contained two transmembrane domains, consistent with the typical membrane topology of plant MGT/MRS2, or CorA-like, magnesium transporters (Table 1).

Secondary structure prediction showed that SiMGT proteins were mainly composed of α-helices, accounting for 50.88–60.36% of the protein sequences, followed by random coils, which accounted for 29.02–37.50%. In contrast, extended strands and β-turns represented smaller proportions, accounting for 6.22–8.55% and 2.53–3.37%, respectively (Table 2). The predominance of α-helices was consistent with the membrane-associated characteristics of SiMGT proteins.

Predicted subcellular localization suggested that SiMGT proteins may function in different membrane systems or organelles. SiMGT1, SiMGT2 and SiMGT4 were mainly predicted to localize to the plasma membrane, whereas SiMGT3 and SiMGT5 showed dominant chloroplast localization signals. SiMGT6 was mainly predicted to localize to the Golgi apparatus, with an additional plasma membrane tendency (Table 2). These results indicate that SiMGT members may have undergone functional diversification and may contribute to Mg^2+^ transport and homeostasis in different cellular compartments.

### 3.3. Phylogenetic Relationship and Conserved Sequence Features of SiMGT Proteins

**Figure 2:**
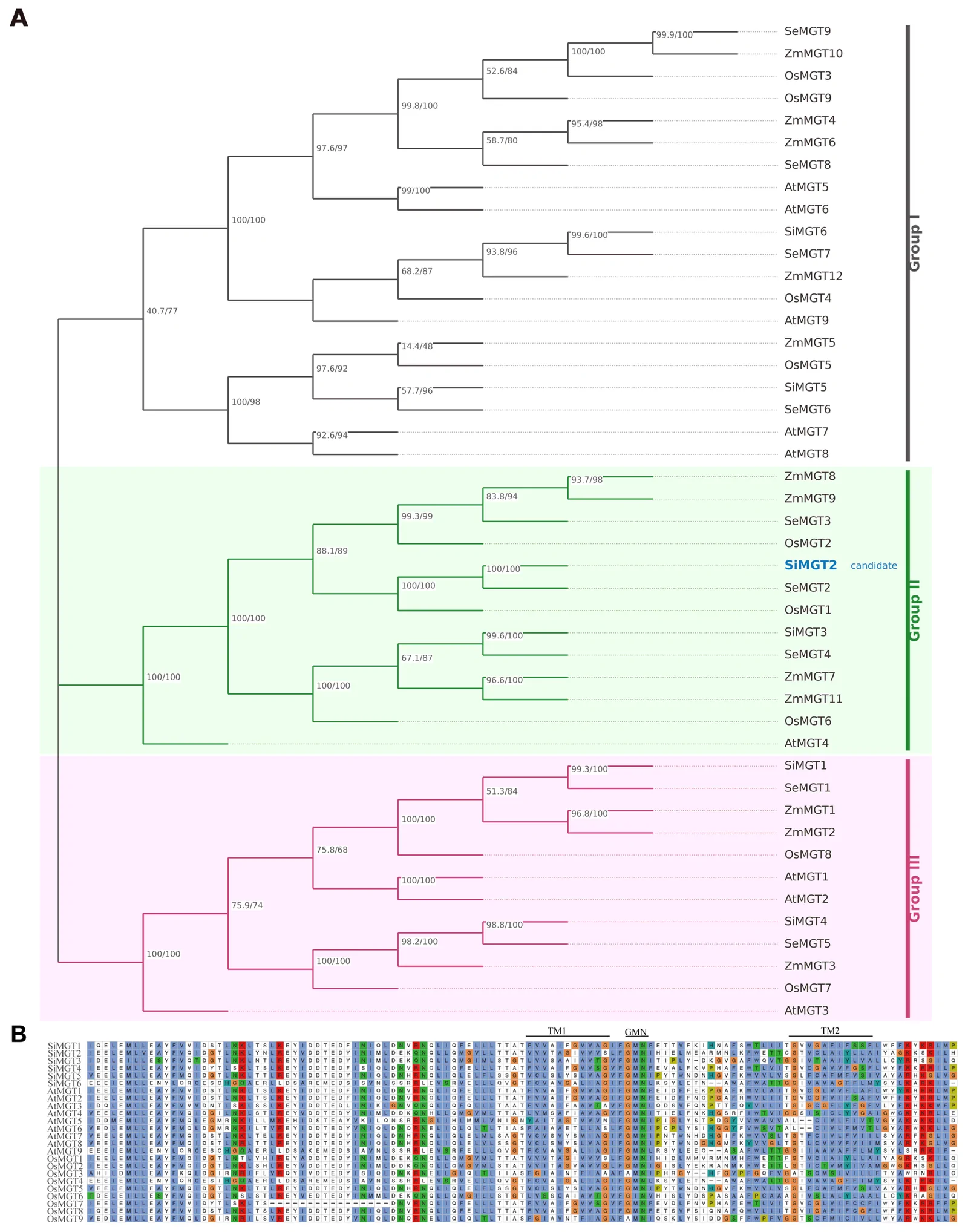
Maximum-likelihood phylogenetic analysis and conserved core sequence alignment of MGT family proteins. (**A**). Maximum-likelihood phylogeny of 45 MGT proteins from foxtail millet, green foxtail, rice, maize and *Arabidopsis*. Branch labels indicate SH-aLRT/UFBoot support values (%) based on 1000 replicates. The three major groups are shown in different colors, and SiMGT2 is highlighted in blue. Branch lengths are not drawn to scale. (**B**) Multiple sequence alignment of the conserved core regions of MGT proteins from foxtail millet, *Arabidopsis* and rice, showing TM1, the conserved GMN motif and TM2. Amino acid residues are colored according to the default ClustalX residue-coloring scheme.

The maximum-likelihood analysis of 45 MGT proteins under JTT+G4 grouped the six SiMGT proteins into three displayed groups: SiMGT5 and SiMGT6 in Group I, SiMGT2 and SiMGT3 in Group II, and SiMGT1 and SiMGT4 in Group III (Figure 2A). SiMGT2 was paired with SeMGT2 with support of 100/100 and joined OsMGT1 with support of 100/100 (SH-aLRT/ultrafast bootstrap, %). These supported sequence relationships inform homology-based comparisons but do not demonstrate identical physiological functions. Some deeper branches had lower support, so the displayed groups are used descriptively.

Multiple sequence alignment was further performed using the conserved core regions of MGT proteins from foxtail millet, *Arabidopsis* and rice (Figure 2B). The results showed that all six SiMGT proteins contained two conserved transmembrane regions, designated as TM1 and TM2. A highly conserved GMN motif was also identified between these two transmembrane regions and was aligned with the corresponding motif in *Arabidopsis* and rice MGT proteins. In addition, several amino acid residues surrounding the GMN motif and the two transmembrane regions were highly conserved among the analyzed MGT proteins, whereas other positions showed sequence variation. These results indicate that SiMGT proteins retain the characteristic conserved structural features of plant MGT family proteins.

### 3.4. Gene Structure, Conserved Motifs and Promoter Cis-Elements of SiMGT Genes

**Figure 3:**
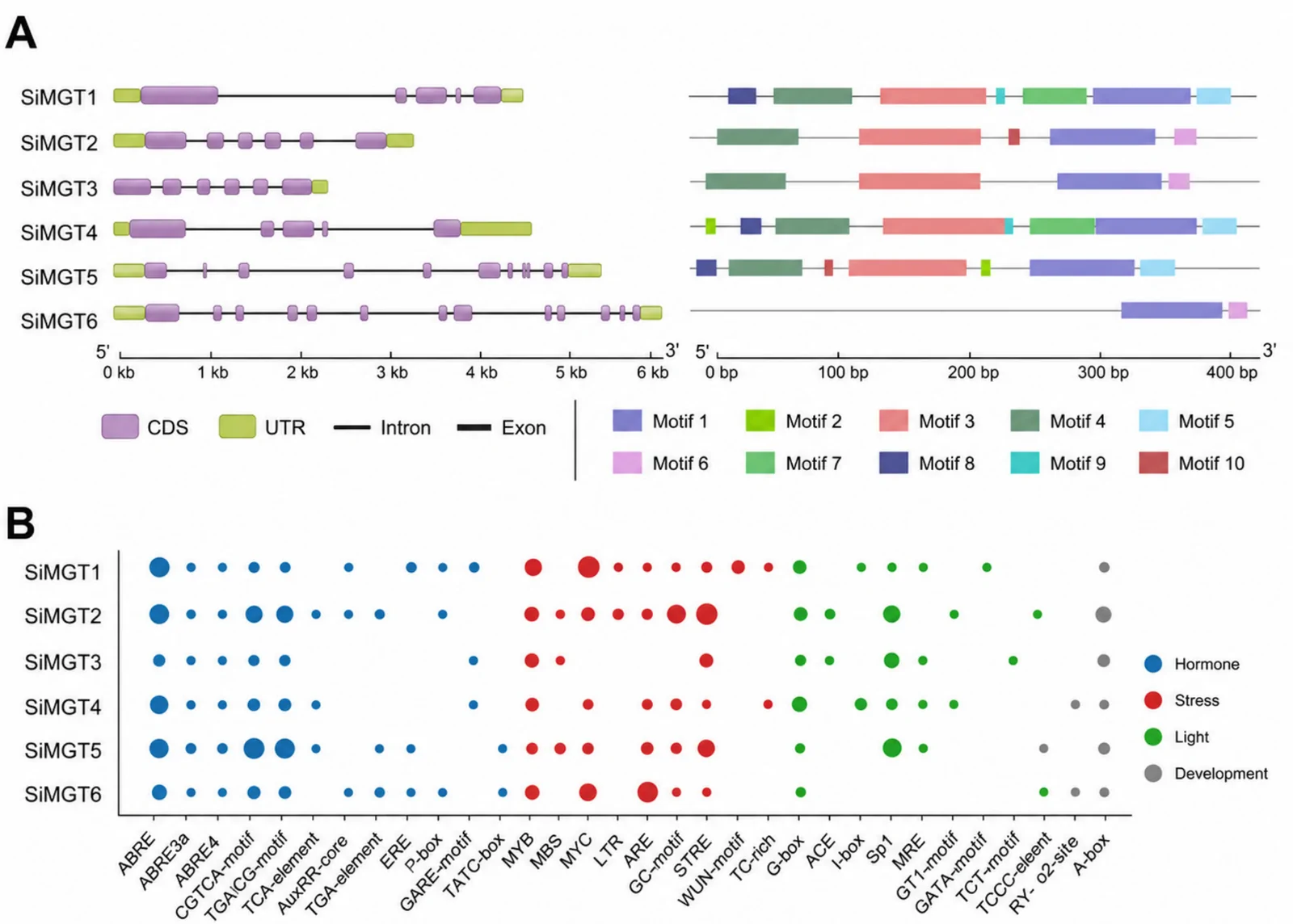
Gene structure, conserved motifs and promoter cis-acting elements of *SiMGT* genes. (**A**) Exon–intron structures and conserved motif compositions of six *SiMGT* genes. Exons, introns, untranslated regions and conserved motifs are indicated by different graphical elements and colors. (**B**) Distribution of cis-acting regulatory elements in the promoter regions of *SiMGT* genes. The identified elements were classified into four major categories, including hormone-responsive, stress-responsive, light-responsive and development-related elements. In panel (**B**), circle size is proportional to the number of predicted cis-acting elements of each type in the promoter region of each *SiMGT* gene.

Gene structure analysis showed that the six *SiMGT* genes differed in gene length and exon–intron organization. *SiMGT1*, *SiMGT2*, *SiMGT4* and *SiMGT5* had relatively short genomic lengths, whereas *SiMGT6* showed the longest gene structure among the six members (Figure 3A). The exon and intron numbers varied among *SiMGT* genes, indicating structural differences within the family.

Conserved motif analysis identified ten motifs in SiMGT proteins. Several motifs were shared by most SiMGT members, including Motif 3, Motif 4, Motif 5 and Motif 7, whereas some motifs showed member-specific distribution patterns. SiMGT1 and SiMGT2 displayed similar motif arrangements, while SiMGT6 contained a relatively extended motif composition compared with the other members (Figure 3A; Table S4). These results showed that SiMGT proteins possess both common conserved motifs and member-specific motif variation.

PlantCARE identified sequence matches to putative hormone-, stress-, light- and development-related cis-elements in the 2000 bp upstream regions of the six *SiMGT* genes (Figure 3B). The annotations included ABRE, MeJA-, auxin- and gibberellin-related motifs, as well as MYB, MBS, MYC, LTR, ARE, STRE, WUN-motif and TC-rich sequences. These categories describe database-annotated sequence features; motif occurrence alone does not establish transcription factor binding, stimulus responsiveness or regulatory activity and does not explain the observed parental expression differences.

### 3.5. Synteny and Evolutionary Rate Analyses of SiMGT Genes

The six *SiMGT* genes were unevenly distributed on Chr4, Chr5, Chr7 and Chr9. Chr7 and Chr9 each contained two *SiMGT* genes, whereas Chr4 and Chr5 each contained one gene. No obvious gene cluster was observed. Intragenomic synteny analysis detected only a limited number of collinear or duplicated relationships involving *SiMGT* genes in the foxtail millet genome (Figure S3A). These relationships involved only a subset of *SiMGT* members, whereas the remaining genes showed no obvious duplicated links in the genome-wide synteny map.

Cross-species synteny analysis identified conserved collinear relationships of *MGT* genes among foxtail millet, green foxtail and rice. More MGT-associated syntenic links were observed between foxtail millet and green foxtail than between foxtail millet and rice. Several *SiMGT* genes showed clear orthologous correspondence with MGT genes in green foxtail and rice, whereas some members showed fewer or no obvious syntenic matches (Figure S3B).

For the selected *SiMGT* paralogous pairs, Ka ranged from 0.214 to 1.116, Ks from 1.971 to 4.306 and Ka/Ks from 0.085 to 0.397. For the foxtail millet–rice homologous pairs, Ka ranged from 0.016 to 0.192, Ks from 0.508 to 0.883 and Ka/Ks from 0.032 to 0.220 (Table 3). Ratios below 1 are consistent with purifying constraints, subject to alignment and substitution model assumptions. The high Ks values of several paralogous pairs could reflect synonymous-site saturation. The rate-dependent times in Table 3 are, therefore, illustrative conversions and are not interpreted as precise evolutionary dates.

### 3.6. Expression Patterns of SiMGT Genes During Panicle Development in Jingu21 and Chuang29

To investigate the expression patterns of *SiMGT* genes in the two parental lines of the RIL population, panicle RNA-seq data from Jingu21 and Chuang29 were analyzed at 7, 14 and 21 days after heading, designated as P-P, P-M and P-L, respectively. Gene expression was quantified as FPKM, and the values were transformed as log_2_(FPKM + 1) for visualization and statistical comparison. Benjamini–Hochberg correction was applied across all 18 gene–stage comparisons (six genes × three stages); q < 0.05 defined statistical significance (Table S5).

**Figure 4:**
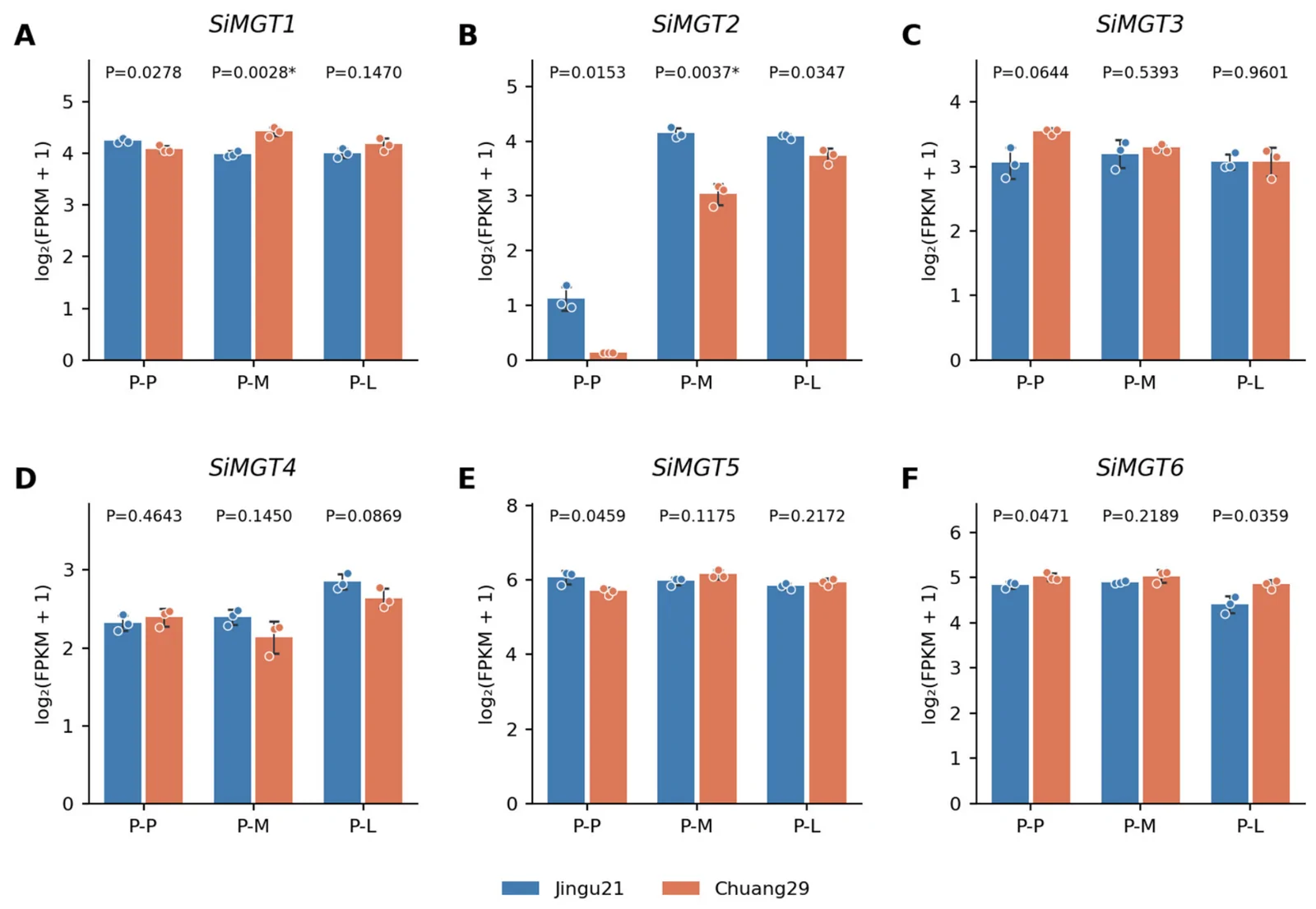
Parental expression comparisons for six *SiMGT* genes. (**A**–**F**) *SiMGT1*–*SiMGT6* expression in Jingu21 and Chuang29 panicles at P-P, P-M and P-L, approximately 7, 14 and 21 days after heading. Bars show means of log_2_(FPKM + 1), error bars show standard deviations, and dots show the three independent biological replicates. Labels show nominal two-sided Welch’s *t*-test *p*-values. An asterisk identifies comparisons significant at q < 0.05 after Benjamini–Hochberg correction across all 18 tests.

The six *SiMGT* genes exhibited distinct genotype- and stage-associated expression patterns during panicle development (Figure 4; Table S5). *SiMGT1* showed significantly higher expression in Jingu21 than in Chuang29 at P-P at the nominal level, whereas its expression was significantly lower in Jingu21 at P-M; after correction, only the P-M difference remained significant (q = 0.0337), while P-P did not reach the adjusted threshold. No significant difference between the two parental lines was detected at P-L.

Notably, the *qFC5*-co-localized candidate gene *SiMGT2* had higher mean expression in Jingu21 at all three sampling times, with nominal *p*-values of 0.0153, 0.0037 and 0.0347 for P-P, P-M and P-L, respectively. After Benjamini–Hochberg correction across the 18 gene–stage comparisons, only its P-M difference remained significant (q = 0.0337; P-P q = 0.0919; P-L q = 0.1059). Thus, while *SiMGT2* showed consistently higher expression in Jingu21 across panicle development, the corrected analysis supports its most pronounced differential expression at the mid-stage (P-M).

The RNA-seq data quality and mapping statistics are summarized in Table S6. Raw reads per sample ranged from 36.4 to 44.8 million, with Q30 values ranging from 94.69% to 95.11%, and mapping rates against the *Setaria italica* v2.2 reference genome ranged from 94.47% to 97.91%, indicating consistently high sequencing quality and reliable read alignment across all 18 libraries. Exploratory sample-level PCA based on unambiguous, variable genes in the FPKM matrix explained 66.79% and 12.27% of the variance on PC1 and PC2, respectively; Jingu21 and Chuang29 samples were clearly separated along PC1, and biological replicates within each genotype–stage group showed good clustering (Figure S5). The Pearson correlation heatmap further confirmed these patterns: biological replicates within the same group exhibited high correlations (r > 0.98), whereas correlations between genotypes were substantially lower, consistent with the PCA results (Figure S5B). The complete FPKM matrix for all expressed genes is provided in Table S7. This processed expression check does not replace inspection of raw-read quality or count-based modeling.

The parental expression comparison supports candidate prioritization but does not establish causality. The RNA-seq plants were grown independently in 2025, whereas the *QTL* phenotype was measured in 2022. Consequently, these datasets do not directly link *SiMGT2* expression to the *qFC5* effect in the same plants, season or environment.

### 3.7. Expression Profiles of SiMGT Genes Across Tissues and Developmental Stages

To further characterize the broader expression patterns of the *SiMGT* family, publicly available foxtail millet transcriptome data from multiple tissues and developmental stages were analyzed. The six *SiMGT* genes displayed divergent tissue- and stage-associated expression profiles, indicating potential functional differentiation among family members (Figure S4). In particular, *SiMGT2* showed detectable expression in multiple tissues and developmental stages, suggesting that its transcriptional activity is not restricted to developing panicles.

### 3.8. Haplotype Analysis of the Candidate Gene SiMGT2

**Figure 5:**
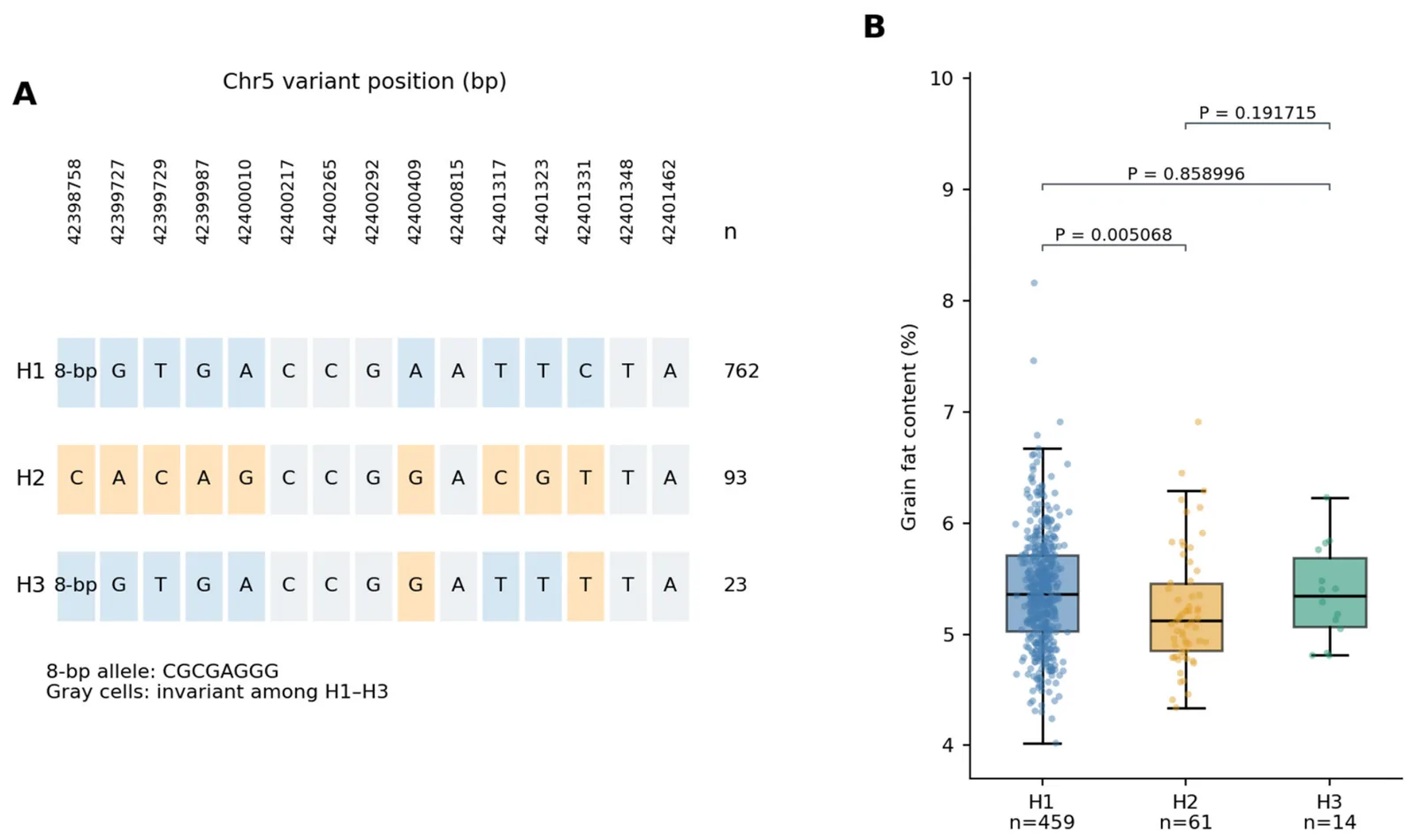
*SiMGT2* haplotype definitions and grain fat content. (**A**) Allele matrix at the 15 displayed chromosome 5 sites. Blue and orange cells indicate alternative allelic states at polymorphic sites, whereas gray cells indicate alleles invariant among H1, H2 and H3. (**B**) Fat content in 534 accessions with 2013_TY phenotypes; sample sizes are shown below the group labels. Blue, orange and green indicate H1, H2 and H3, respectively. Boxes show medians and interquartile ranges, whiskers extend to 1.5 times the interquartile range, and dots show individual accessions. Labels report nominal two-sided Wilcoxon rank-sum *p*-values; the H1–H2 comparison also meets BH-FDR q < 0.05.

The supplied *SiMGT2* variant display contained 15 sites: one indel and 14 single-nucleotide sites. Nine sites distinguished H1, H2 and H3, while six had the same allele in all three groups (Figure 5A; Table S8). There were 762, 93 and 23 assigned accessions, respectively (N = 878), corresponding to frequencies of 86.79%, 10.59% and 2.62%. H1 and H3 differed at Chr5:42,400,409 and Chr5:42,401,331. The former lies in the annotated coding region; predicted variant consequences are annotations rather than experimentally established functional effects.

The phenotype analysis comprised 534 accessions: H1, *n* = 459; H2, *n* = 61; and H3, *n* = 14. The mean grain fat contents were 5.39%, 5.22% and 5.36%, respectively. H1 exceeded H2 by 0.1655 percentage points (95% CI: 0.0231–0.3078; nominal *p* = 0.0051; q = 0.0152). H1 versus H3 and H2 versus H3 were not significant (nominal *p* = 0.8589 and 0.1917; q = 0.8589 and 0.2876, respectively). Because the comparison was limited to 2013_TY, had unequal group sizes and did not account for population structure or kinship, H1 is described as a putatively favorable haplotype associated with a higher fat content than H2 in this dataset. This does not establish a causal or broadly transferable haplotype effect (Figure 5B; Table S8).

## 4. Discussion

### 4.1. Genetic Dissection of Grain Fat Content Provides Information for Lipid-Related Grain Composition in Foxtail Millet

Grain chemical composition is an important target for value-added utilization and germplasm improvement in foxtail millet [2,3]. Grain fat content is a lipid-related component that contributes to the chemical profile, energy value and processing-related properties of millet grains, whereas the genetic basis of this trait remains less well characterized than that of major agronomic and yield-related traits [5,8,9,10,11]. Therefore, identifying genomic regions and candidate genes associated with grain fat content is important for expanding genetic resources for lipid-related grain composition improvement and value-added utilization.

In the present study, *qFC5* was detected in 2022_SY (LOD: 5.86; PVE: 9.24%) and supported by an overlapping BLUE-based interval (LOD: 5.99; PVE: 7.67%). The locus, therefore, has environment-dependent detectability and has not been established as a stable breeding *QTL*. The significant environmental effect and modest inter-environment correlation show that performance differed across trials, but neither these observations nor differences in *QTL* significance constitute a formal genotype × environment or *QTL* × environment test. Unequal numbers of valid grain samples and the use of line mean limited separation of interaction and residual variation. Further multi-environment testing with retained plot-level observations is required.

The overlapping *QTL* intervals provide a starting region for candidate gene evaluation. Their breadth and different peak markers prevent attribution of the association to a single gene or variant. Homology-informed annotation and expression information can focus subsequent experiments, but other genes within the interval remain possible contributors. *qFC5* and *SiMGT2* should, therefore, be regarded as targets for further genetic validation.

### 4.2. Integrated Evidence Prioritizes SiMGT2 as a Candidate Gene for Grain Fat Content

Candidate gene prioritization combined the 248-gene interval catalog with *Arabidopsis* and rice homolog annotations and developmental expression context. *SiMGT2* was selected as a plausible magnesium transporter candidate; its MGT/MRS2 domain, conserved core sequence and supported phylogenetic relationships provide annotation context [18,19,49]. The family analysis characterizes the selected candidate and its relatives but does not independently demonstrate a grain fat function. The present evidence does not uniquely rank *SiMGT2* above every other interval gene.

The H1–H2 difference was small in absolute terms and remained significant after correction for three comparisons, but neither comparison involving H3 was significant. The H3 sample comprised only 14 phenotyped accessions, limiting precision and power. The earlier population structure and GWAS analyses of the source panel do not adjust for the present haplotype comparison [9]. Ancestry, relatedness and other linked loci could confound the observed group difference. H1 is, therefore, putatively favorable only in the limited descriptive sense of higher fat content than H2 in 2013_TY, pending structure- and kinship-adjusted validation.

Higher mean *SiMGT2* expression in Jingu21, including a significant P-M difference after correction across 18 tests, provides supportive expression information. However, the three-replicate FPKM comparison has limited precision and was conducted in 2025, independently of the 2022 phenotyping. It does not establish a mechanistic expression–phenotype relationship or demonstrate that *SiMGT2* explains the *QTL* association. Joint phenotyping and expression measurements in the same genetic materials and environments, followed by functional perturbation, are needed.

### 4.3. Possible Links Between Magnesium Transport and Grain Fat Accumulation

Mg^2+^ participates in photosynthesis, enzyme activation and Mg-ATP-dependent metabolism [16,17]. These processes provide a physiological rationale for testing whether variation in a predicted magnesium transporter is associated with the allocation of carbon and energy to grain storage compounds. MGT/MRS2 proteins are not established lipid-biosynthetic enzymes, and the pathway linking *SiMGT2* to grain fat content remains hypothetical.

Reported effects of Mg nutrition on rapeseed carbon assimilation and seed composition [50] provide general physiological context and cannot establish a *SiMGT2* mechanism in foxtail millet. Grain Mg concentration, *SiMGT2* transport activity and fatty acid composition were not measured in this study. It is, therefore, unknown whether the candidate affects grain fat through Mg homeostasis, carbon assimilation, Mg-ATP-dependent metabolism or another process. Coordinated Mg and lipid measurements in parents, selected RILs and functionally validated *SiMGT2* materials are needed to test these alternatives.

The evidence should be distinguished at three levels: *QTL* mapping associates a genomic interval with the phenotype; homolog annotation, expression and exploratory haplotype comparisons prioritize a candidate within that interval; and functional experiments would be needed to establish causality. Gene editing, complementation or near-isogenic materials, together with grain Mg and lipid measurements, are required before a causal mechanism or breeding application can be proposed.

## 5. Conclusions

An environment-dependent chromosome 5 grain-fat *QTL* was supported by 2022_SY and across-environment BLUE analyses. Among 248 genes in the Sanya interval, homology, annotation and expression information prioritized *SiMGT2* as a candidate gene. The MGT/MRS2 family analysis provides genomic context, while the parental expression and unadjusted-for-structure haplotype comparisons provide supportive, associative information. Neither causality nor a connection between *SiMGT2*-mediated Mg homeostasis and lipid accumulation has been established. Functional validation and coordinated grain Mg and lipid measurements are essential.

## Figures and Tables

**Table 1:** Basic characteristics of *SiMGT* genes in foxtail millet.

Gene ID	Gene Name	Chr	Start	End	Length (aa)	MW (kDa)	pI	TM Domain
*SiMGT1*	*Seita.4G195900.1*	Chr4	31,380,962	31,385,091	430	47.83	5.03	2
*SiMGT2*	*Seita.5G394800.1*	Chr5	42,398,706	42,401,512	411	45.64	4.75	2
*SiMGT3*	*Seita.7G099600.1*	Chr7	20,256,768	20,258,700	407	43.51	4.47	2
*SiMGT4*	*Seita.7G156200.1*	Chr7	24,116,053	24,120,424	435	48.13	5.16	2
*SiMGT5*	*Seita.9G097500.1*	Chr9	5,865,418	5,870,918	387	42.70	4.61	2
*SiMGT6*	*Seita.9G138000.1*	Chr9	8,750,707	8,757,024	457	50.07	5.08	2

Note: MW, molecular weight of protein; pI, protein isoelectric point.

**Table 2:** Secondary structure and predicted subcellular localization of SiMGT proteins.

Gene ID	Alpha Helix (%)	Extended Strand (%)	Beta Turn (%)	Random Coil (%)	Subcellular Localization
*SiMGT1*	54.31	6.29	2.56	36.83	plas (7); E.R. (3)
*SiMGT2*	59.27	6.59	3.17	30.98	plas (5.5); E.R. (3)
*SiMGT3*	58.37	7.64	2.71	31.28	chlo (6); E.R. (4)
*SiMGT4*	54.38	6.22	2.53	36.87	plas (9); vacu (2)
*SiMGT5*	60.36	7.25	3.37	29.02	chlo (3); E.R. (3)
*SiMGT6*	50.88	8.55	3.07	37.50	golg (4); plas (3)

Note: plas: plasma membrane; E.R.: endoplasmic reticulum; chlo: chloroplast; golg: Golgi apparatus; vacu: vacuole.

**Table 3:** Evolutionary rates of duplicated and orthologous *MGT* gene pairs in foxtail millet and rice.

Gene Pair		Ka	Ks	Ka/Ks	MYA	*p*-Value	Aligned Length (bp)
*SiMGT5*	*SiMGT6*	1.116	2.814	0.397	154.64	4.10 × 10^−6^	1032
*SiMGT3*	*SiMGT4*	0.442	3.951	0.112	217.09	1.23 × 10^−34^	1095
*SiMGT4*	*SiMGT1*	0.214	1.971	0.109	108.28	2.13 × 10^−56^	1275
*SiMGT2*	*SiMGT3*	0.343	4.051	0.085	222.56	8.71 × 10^−48^	1176
*SiMGT6*	*SiMGT4*	0.941	4.306	0.219	236.59	8.48 × 10^−14^	1140
*SiMGT1*	*OsMGT8*	0.047	0.721	0.065	39.61	7.75 × 10^−57^	1284
*SiMGT2*	*OsMGT1*	0.165	0.883	0.187	48.52	3.79 × 10^−24^	1122
*SiMGT3*	*OsMGT6*	0.192	0.873	0.220	47.95	2.52 × 10^−21^	1188
*SiMGT5*	*OsMGT5*	0.016	0.508	0.032	27.90	1.03 × 10^−33^	747

Note: Ka and Ks denote the nonsynonymous and synonymous substitution rates, respectively. MYA indicates million years ago.

## Data Availability

The data presented in this study are available within the article and its Supplementary Materials. The original QTL mapping data and RNA-seq datasets generated in this study are available from the corresponding authors upon reasonable request.

## Supplementary Materials

The following supporting information can be downloaded at https://www.mdpi.com/article/10.3390/biology15181631/s1. Table S1. Grain fat content of the Jingu21 × Chuang29 RIL population across environments; Table S2. *QTL* detected for grain fat content in the Jingu21 × Chuang29 RIL population; Table S3. Detailed information for annotated genes located within the *qFC5* interval; Table S4. MEME-predicted motifs of the SiMGT protein family in foxtail millet; Table S5. Expression levels of six *SiMGT* genes in panicles of Jingu21 and Chuang29 at three developmental stages; Table S6. Summary of RNA-seq data quality and mapping statistics; Table S7. FPKM expression matrix of *SiMGT* genes in JG21 and Chuang29 panicles at three developmental stages; Table S8. Grain fat content and *SiMGT2* haplotypes in the germplasm panel; Figure S1. Phenotypic variation in grain fat content in the Jingu21 × Chuang29 RIL population across two environments; Figure S2. Genome-wide LOD profile for grain fat content in 2022_YC environment; Figure S3. Synteny relationships of *MGT* genes in foxtail millet and related grass species; Figure S4. Developmental and tissue-specific expression patterns of *SiMGT* genes in foxtail millet; Figure S5. PCA and sample correlation heatmap of RNA-seq samples from parental lines JG21 and Chuang29 at three panicle developmental stages.

## References

[1] Peng R., Zhang B. (2021). Foxtail millet: A new model for C4 plants. Trends Plant Sci..

[2] Diao X., Schnable J., Bennetzen J., Li J. (2014). Initiation of Setaria as a model plant. Front. Agric. Sci. Eng..

[3] Doust A.N., Kellogg E.A., Devos K.M., Bennetzen J.L. (2009). Foxtail millet: A sequence-driven grass model system. Plant Physiol..

[4] Yang T., Ma S., Liu J., Sun B., Wang X. (2022). Influences of four processing methods on main nutritional components of foxtail millet: A review. Grain Oil Sci. Technol..

[5] Abedin M.J., Abdullah A.T.M., Satter M.A., Farzana T. (2022). Physical, functional, nutritional and antioxidant properties of foxtail millet in Bangladesh. Heliyon.

[6] Kalsi R., Bhasin J.K. (2023). Nutritional exploration of foxtail millet (*Setaria italica*) in addressing food security and its utilization trends in food system. eFood.

[7] Garutti M., Nevola G., Mazzeo R., Cucciniello L., Totaro F., Bertuzzi C.A., Caccialanza R., Pedrazzoli P., Puglisi F. (2022). The impact of cereal grain composition on the health and disease outcomes. Front. Nutr..

[8] Shahidi F., Hossain A. (2022). Role of lipids in food flavor generation. Molecules.

[9] Jia G., Huang X., Zhi H., Zhao Y., Zhao Q., Li W., Chai Y., Yang L., Liu K., Lu H. (2013). A haplotype map of genomic variations and genome-wide association studies of agronomic traits in foxtail millet (*Setaria italica*). Nat. Genet..

[10] Jaiswal V., Gupta S., Gahlaut V., Muthamilarasan M., Bandyopadhyay T., Ramchiary N., Prasad M. (2019). Genome-wide association study of major agronomic traits in foxtail millet (*Setaria italica* L.) using ddRAD sequencing. Sci. Rep..

[11] He Q., Tang S., Zhi H., Chen J., Zhang J., Liang H., Alam O., Li H., Zhang H., Xing L. (2023). A graph-based genome and pan-genome variation of the model plant Setaria. Nat. Genet..

[12] He Q., Zhi H., Tang S., Xing L., Wang S., Wang H., Zhang A., Li Y., Gao M., Zhang H. (2021). QTL mapping for foxtail millet plant height in multi-environment using an ultra-high density bin map. Theor. Appl. Genet..

[13] Wei W., Li S., Li P., Yu K., Fan G., Wang Y., Zhao F., Zhang X., Feng X., Shi G. (2023). QTL analysis of important agronomic traits and metabolites in foxtail millet (*Setaria italica*) by RIL population and widely targeted metabolome. Front. Plant Sci..

[14] Zhang L., Ma K., Zhao X., Li Z., Zhang X., Li W., Meng R., Lu B., Yuan X. (2023). Development of a comprehensive quality evaluation system for foxtail millet from different ecological regions. Foods.

[15] Ma K., Zhao X., Lu B., Wang Y., Yue Z., Zhang L., Diao X., Yuan X. (2024). Effect of ecological factors on nutritional quality of foxtail millet (*Setaria italica* L.). Agronomy.

[16] Kobayashi N.I., Tanoi K. (2015). Critical issues in the study of magnesium transport systems and magnesium deficiency symptoms in plants. Int. J. Mol. Sci..

[17] Kleczkowski L.A., Igamberdiev A.U. (2021). Magnesium signaling in plants. Int. J. Mol. Sci..

[18] Li L., Tutone A.F., Drummond R.S., Gardner R.C., Luan S. (2001). A novel family of magnesium transport genes in Arabidopsis. Plant Cell.

[19] Saito T., Kobayashi N.I., Tanoi K., Iwata N., Suzuki H., Iwata R., Nakanishi T.M. (2013). Expression and functional analysis of the CorA-MRS2-ALR-type magnesium transporter family in rice. Plant Cell Physiol..

[20] Conn S.J., Conn V., Tyerman S.D., Kaiser B.N., Leigh R.A., Gilliham M. (2011). Magnesium transporters, MGT2/MRS2-1 and MGT3/MRS2-5, are important for magnesium partitioning within Arabidopsis thaliana mesophyll vacuoles. New Phytol..

[21] Drummond R., Tutone A., Li Y.-C., Gardner R. (2006). A putative magnesium transporter AtMRS2-11 is localized to the plant chloroplast envelope membrane system. Plant Sci..

[22] Chen Y., Zhang W., Zhao F., Liu G., Zhao D., Xu J., Wang X., Zong X., Zhang J., Ji X. (2025). Genome-Wide Identification, Phylogeny and Expression Analysis of the Magnesium Release Gene Family in Wheat (*Triticum aestivum* L.). Curr. Issues Mol. Biol..

[23] Zhang L., Wen A., Wu X., Pan X., Wu N., Chen X., Chen Y., Mao D., Chen L., Luan S. (2019). Molecular identification of the magnesium transport gene family in Brassica napus. Plant Physiol. Biochem..

[24] Liu W., Khan S., Tong M., Hu H., Yin L., Huang J. (2023). Identification and Expression of the CorA/MRS2/ALR Type Magnesium Transporters in Tomato. Plants.

[25] Zhou W., Zhi H., Liu B., He Q., Sun Y., Wang L., Xie H., Gao M., Yang Q., Wang H. (2026). QTL dissection of yield-related traits in foxtail millet using cross-ecotype RIL. J. Integr. Agric..

[26] Mistry J., Chuguransky S., Williams L., Qureshi M., Salazar G.A., Sonnhammer E.L., Tosatto S.C., Paladin L., Raj S., Richardson L.J. (2021). Pfam: The protein families database in 2021. Nucleic Acids Res..

[27] Finn R.D., Clements J., Eddy S.R. (2011). HMMER web server: Interactive sequence similarity searching. Nucleic Acids Res..

[28] Berardini T.Z., Reiser L., Li D., Mezheritsky Y., Muller R., Strait E., Huala E. (2015). The Arabidopsis information resource: Making and mining the “gold standard” annotated reference plant genome. Genesis.

[29] Camacho C., Coulouris G., Avagyan V., Ma N., Papadopoulos J., Bealer K., Madden T.L. (2009). BLAST+: Architecture and applications. BMC Bioinform..

[30] Goodstein D.M., Shu S., Howson R., Neupane R., Hayes R.D., Fazo J., Mitros T., Dirks W., Hellsten U., Putnam N. (2012). Phytozome: A comparative platform for green plant genomics. Nucleic Acids Res..

[31] Hallgren J., Tsirigos K.D., Pedersen M.D., Almagro Armenteros J.J., Marcatili P., Nielsen H., Krogh A., Winther O. (2022). DeepTMHMM predicts alpha and beta transmembrane proteins using deep neural networks. bioRxiv.

[32] Wickham H. (2016). Data analysis. ggplot2: Elegant Graphics for Data Analysis.

[33] Osorio D., Rondón-Villarreal P., Torres R. (2015). Peptides: A package for data mining of antimicrobial peptides. R J..

[34] Geourjon C., Deleage G. (1995). SOPMA: Significant improvements in protein secondary structure prediction by consensus prediction from multiple alignments. Bioinformatics.

[35] Horton P., Park K.-J., Obayashi T., Fujita N., Harada H., Adams-Collier C., Nakai K. (2007). WoLF PSORT: Protein localization predictor. Nucleic Acids Res..

[36] Bodenhofer U., Bonatesta E., Horejš-Kainrath C., Hochreiter S. (2015). msa: An R package for multiple sequence alignment. Bioinformatics.

[37] Sievers F., Wilm A., Dineen D., Gibson T.J., Karplus K., Li W., Lopez R., McWilliam H., Remmert M., Söding J. (2011). Fast, scalable generation of high-quality protein multiple sequence alignments using Clustal Omega. Mol. Syst. Biol..

[38] Vilella A.J., Severin J., Ureta-Vidal A., Heng L., Durbin R., Birney E. (2009). EnsemblCompara GeneTrees: Complete, duplication-aware phylogenetic trees in vertebrates. Genome Res..

[39] Jones D.T., Taylor W.R., Thornton J.M. (1992). The rapid generation of mutation data matrices from protein sequences. Bioinformatics.

[40] Hoang D.T., Chernomor O., Von Haeseler A., Minh B.Q., Vinh L.S. (2018). UFBoot2: Improving the ultrafast bootstrap approximation. Mol. Biol. Evol..

[41] Bailey T.L., Johnson J., Grant C.E., Noble W.S. (2015). The MEME suite. Nucleic Acids Res..

[42] Hu B., Jin J., Guo A.-Y., Zhang H., Luo J., Gao G. (2015). GSDS 2.0: An upgraded gene feature visualization server. Bioinformatics.

[43] Lescot M., Déhais P., Thijs G., Marchal K., Moreau Y., Van de Peer Y., Rouzé P., Rombauts S. (2002). PlantCARE, a database of plant cis-acting regulatory elements and a portal to tools for in silico analysis of promoter sequences. Nucleic Acids Res..

[44] Wang Y., Tang H., DeBarry J.D., Tan X., Li J., Wang X., Lee T.-h., Jin H., Marler B., Guo H. (2012). MCScanX: A toolkit for detection and evolutionary analysis of gene synteny and collinearity. Nucleic Acids Res..

[45] Wang D., Zhang Y., Zhang Z., Zhu J., Yu J. (2010). KaKs_Calculator 2.0: A toolkit incorporating gamma-series methods and sliding window strategies. Genom. Proteom. Bioinform..

[46] Yang Z., Nielsen R. (2000). Estimating synonymous and nonsynonymous substitution rates under realistic evolutionary models. Mol. Biol. Evol..

[47] Luo S., Hu W., Wang Y., Liu B., Yan H., Xiang Y. (2018). Genome-wide identification, classification, and expression of phytocyanins in Populus trichocarpa. Planta.

[48] He Q., Wang C., Zhang J., Liang H., Lu Z., Xie K., Tang S., Zhou Y., Liu B., Zhi H. (2024). A complete reference genome assembly for foxtail millet and Setaria-db, a comprehensive database for Setaria. Mol. Plant.

[49] Li H., Du H., Huang K., Chen X., Liu T., Gao S., Liu H., Tang Q., Rong T., Zhang S. (2016). Identification, and functional and expression analyses of the CorA/MRS2/MGT-type magnesium transporter family in maize. Plant Cell Physiol..

[50] Geng G., Cakmak I., Ren T., Lu Z., Lu J. (2021). Effect of magnesium fertilization on seed yield, seed quality, carbon assimilation and nutrient uptake of rapeseed plants. Field Crops Res..

