# Supplementary material for: QTL-Guided Prioritization of SiMGT2 as a Candidate Gene Associated with Grain Fat Content in Foxtail Millet
Source: Biology (Basel). 2026 Sep 16;15(18):1631. doi: 10.3390/biology15181631 (PMC13604400; doi:10.3390/biology15181631)
Supplement: Supplementary file 1 [file biology-15-01631-s001.zip › Figure S1. Phenotypic variation of grain fat content in the Jingu21 × Chuang29 RIL population across two environments.pdf]

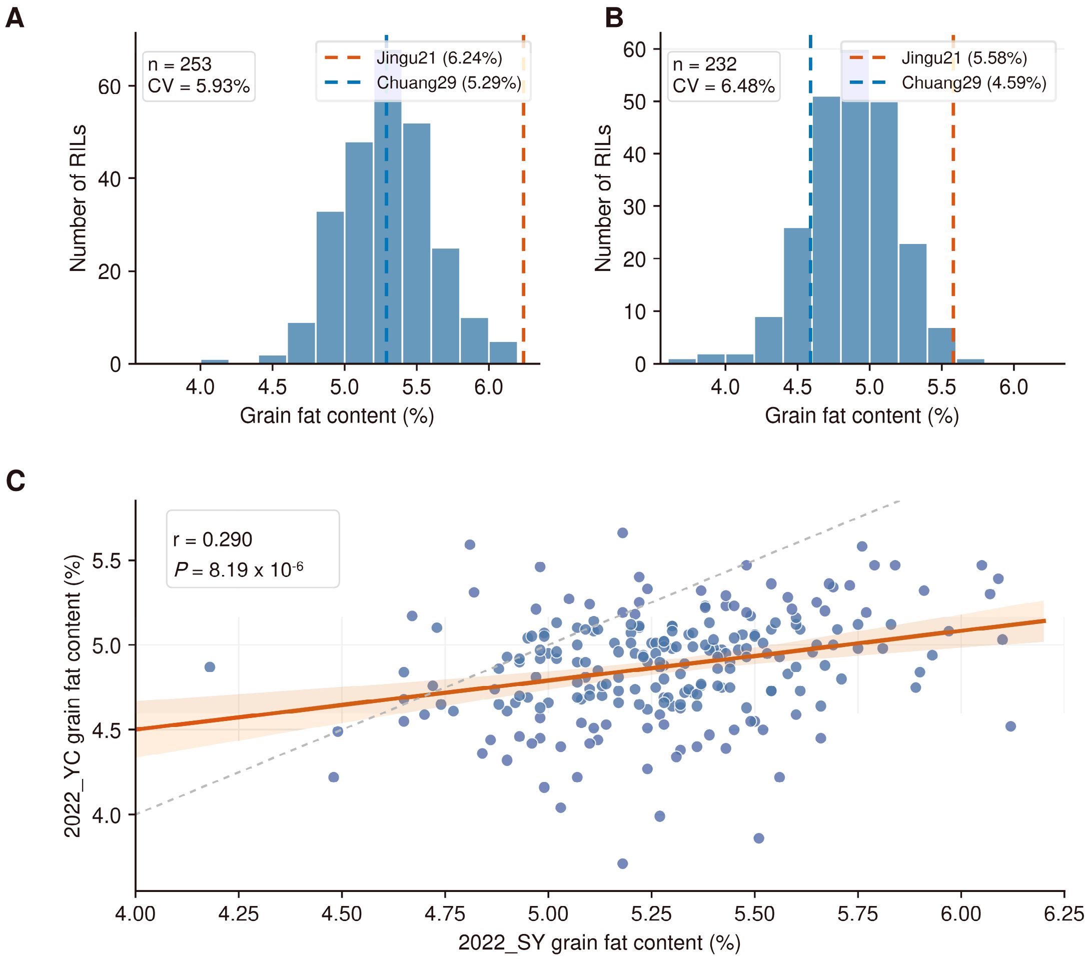

Figure S1. Phenotypic variation of grain fat content in the Jingu21 × Chuang29 RIL population across two environments. (**A**). Frequency distribution of grain fat content in 2022_SY. (**B**). Frequency distribution of grain fat content in 2022_YC. (**C**). Correlation of RIL performance between 2022_SY and 2022_YC based on 229 RILs with complete data. Vertical dashed lines in A and B indicate parental values of Jingu21 and Chuang29. The shaded band in C indicates the 95% confidence interval of the fitted regression line.
