# Supplementary material for: QTL-Guided Prioritization of SiMGT2 as a Candidate Gene Associated with Grain Fat Content in Foxtail Millet
Source: Biology (Basel). 2026 Sep 16;15(18):1631. doi: 10.3390/biology15181631 (PMC13604400; doi:10.3390/biology15181631)
Supplement: Supplementary file 1 [file biology-15-01631-s001.zip › Figure S2. Genome-wide LOD profile for grain fat content in 2022_YC environment.pdf]

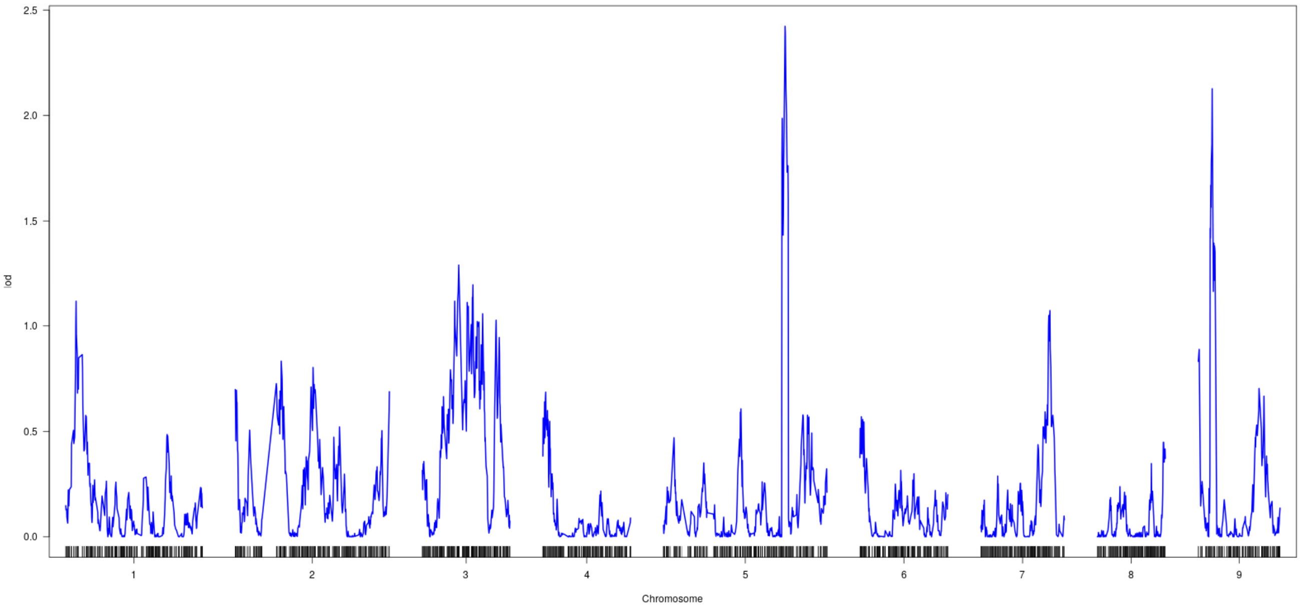

Figure S2. Genome-wide LOD profile for grain fat content in 2022_YC environment.
