# Supplementary material for: QTL-Guided Prioritization of SiMGT2 as a Candidate Gene Associated with Grain Fat Content in Foxtail Millet
Source: Biology (Basel). 2026 Sep 16;15(18):1631. doi: 10.3390/biology15181631 (PMC13604400; doi:10.3390/biology15181631)
Supplement: Supplementary file 1 [file biology-15-01631-s001.zip › Figure S3. Synteny relationships of MGT genes in foxtail millet and related grass species.pdf]

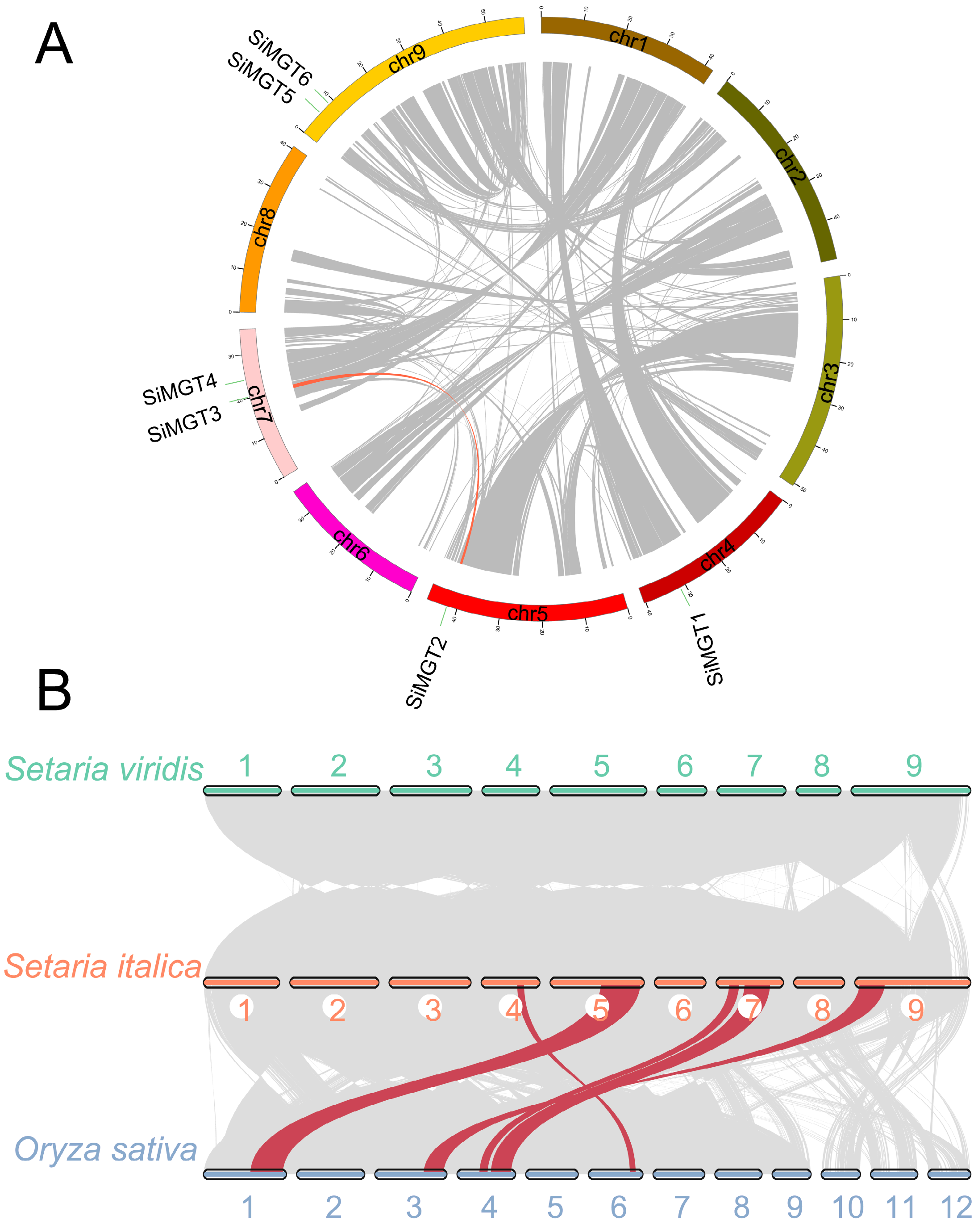

Figure S3. Synteny relationships of *MGT* genes in foxtail millet and related grass species. (**A**). Intragenomic collinearity of *SiMGT* genes in the foxtail millet genome. Grey lines indicate genome-wide collinear blocks, and red lines indicate collinear relationships involving *SiMGT* genes. (**B**). Comparative synteny of *MGT* genes among *Setaria viridis*, *Setaria italica* and *Oryza sativa*. Grey lines represent genome-wide syntenic blocks, and red lines highlight MGT-associated orthologous relationships.
