# Supplementary material for: QTL-Guided Prioritization of SiMGT2 as a Candidate Gene Associated with Grain Fat Content in Foxtail Millet
Source: Biology (Basel). 2026 Sep 16;15(18):1631. doi: 10.3390/biology15181631 (PMC13604400; doi:10.3390/biology15181631)
Supplement: Supplementary file 1 [file biology-15-01631-s001.zip › Figure S4. Developmental and tissue-specific expression patterns of SiMGT genes in foxtail millet.pdf]

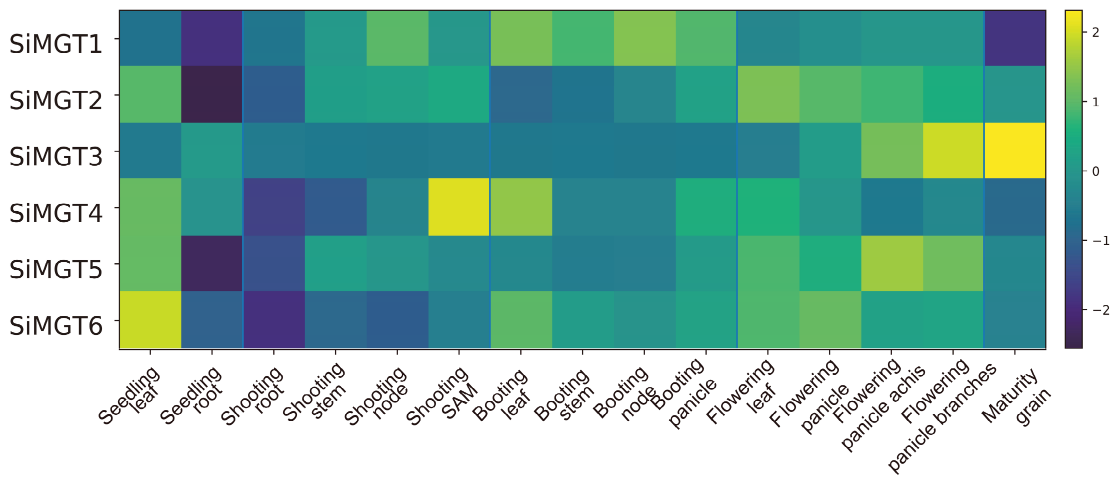

Figure S4. Developmental and tissue-specific expression patterns of *SiMGT* genes in foxtail millet. Expression profiles of six *SiMGT* genes were analyzed across different tissues and developmental stages of foxtail millet. Samples are ordered by developmental progression from seedling to maturity. Expression values were transformed as log2(FPKM + 1) and normalized by row-wise z-scores. The color scale represents relative expression levels within each gene. SAM indicates shoot apical meristem.
