# Supplementary material for: QTL-Guided Prioritization of SiMGT2 as a Candidate Gene Associated with Grain Fat Content in Foxtail Millet
Source: Biology (Basel). 2026 Sep 16;15(18):1631. doi: 10.3390/biology15181631 (PMC13604400; doi:10.3390/biology15181631)
Supplement: Supplementary file 1 [file biology-15-01631-s001.zip › Figure S5. PCA analysis and sample correlation heatmap of RNA-seq samples from parental lines JG21 and Chuang29 at three panicle developmental stages.pdf]

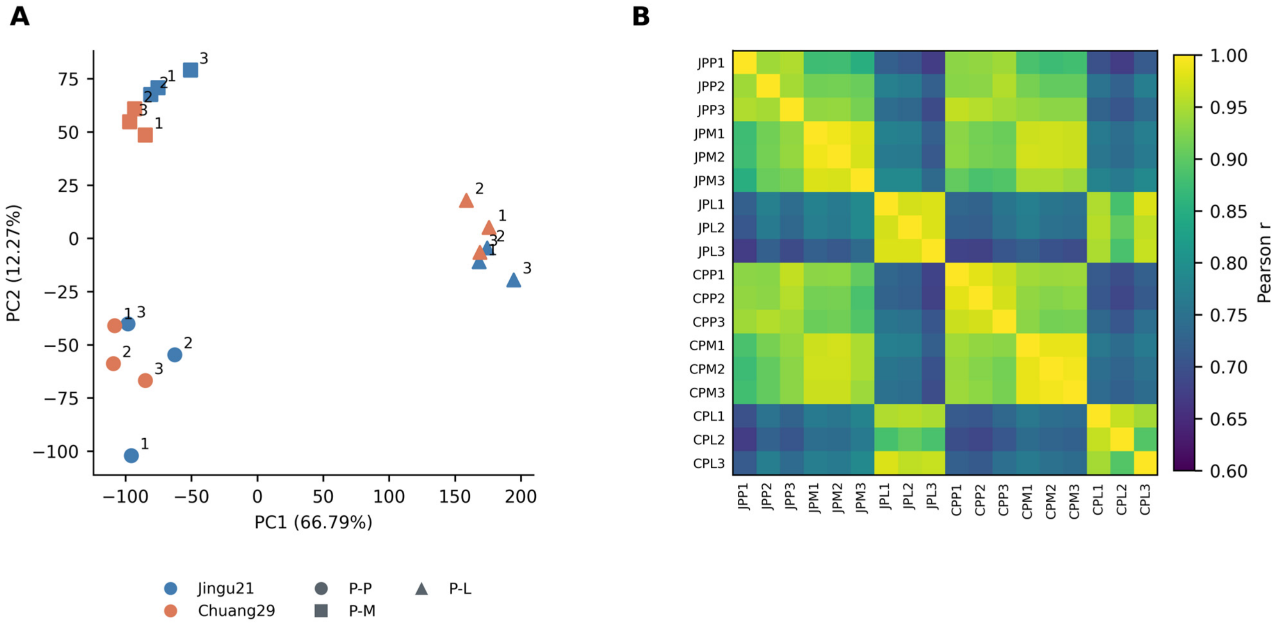

Figure S5. PCA analysis and sample correlation heatmap of RNA-seq samples from parental lines JG21 and Chuang29 at three panicle developmental stages. (**A**) Principal component analysis based on log_2(FPKM + 1) values of all expressed genes. PC1 and PC2 accounted for 66.79% and 12.27% of the total variance, respectively. JG21 samples (red) and Chuang29 samples (blue) were clearly separated along PC1, indicating that genotype is the major source of transcriptomic variation. Three biological replicates are shown for each genotype–stage combination, with good clustering within each group, demonstrating high experimental reproducibility. P-P, P-M and P-L represent panicle samples collected at approximately 7, 14 and 21 days after heading, respectively. (**B**) Heatmap of Pearson correlation coefficients among all samples, calculated based on log_2(FPKM + 1) values of all expressed genes. Color intensity indicates correlation strength (from light for low correlation to dark for high correlation). Biological replicates within the same group showed high positive correlations ($r > 0.98$), further confirming the reliability and reproducibility of the RNA-seq data. Correlations between genotypes (JG21 vs Chuang29) were relatively lower, consistent with the PCA results and supporting substantial transcriptomic divergence between the two parental lines.
